# Mo-doped ZnO nanoflakes on Ni-foam for asymmetric supercapacitor applications†

**DOI:** 10.1039/c9ra05051e

**Published:** 2019-09-02

**Authors:** Awais Ali, Muhammad Ammar, Muddassir Ali, Zaid Yahya, Muhammad Yasar Javaid, Sadaf ul Hassan, Toheed Ahmed

**Affiliations:** Department of Chemical Engineering Technology, Government College University Faisalabad 38000 Pakistan mammar@gcuf.edu.pk; University of Chinese Academy of Sciences Beijing 100049 China; Department of Energy Engineering, Faculty of Mechanical and Aeronautical Engineering, University of Engineering and Technology Taxila 47080 Pakistan; Department of Mechanical Engineering Technology, Government College University Faisalabad 38000 Pakistan; Department of Chemistry, COMSATS University Islamabad Lahore Campus 45550 Pakistan; Department of Applied Chemistry, Government College University Faisalabad 38000 Pakistan

## Abstract

A single-step hydrothermal route for synthesizing molybdenum doped zinc oxide nanoflakes was employed to accomplish superior electrochemical characteristics, such as a specific capacitance of 2296 F g^−1^ at current density of 1 A g^−1^ and negligible loss in specific capacitance of 0.01025 F g^−1^ after each charge–discharge cycle (up to 8000 cycles). An assembled asymmetric supercapacitor (Mo:ZnO@NF//AC@NF) also exhibited a maximum energy density and power density of 39.06 W h/kg and 7425 W kg^−1^, respectively. Furthermore, it demonstrated a specific capacitance of 123 F g^−1^ at 1 A g^−1^ and retained about 75.6% of its initial capacitance after 8000 cycles. These superior electrochemical characteristics indicate the potential of this supercapacitor for next-generation energy storage devices.

## Abstract 2

Mo:ZnO nanoflakes were synthesized by single-step hydrothermal route to achieve superior electrochemical performance.

## Graphical abstract

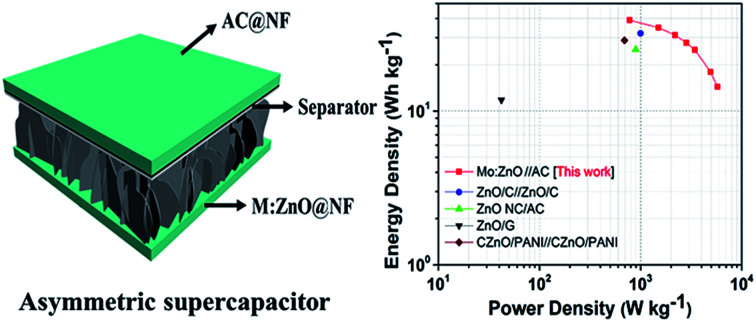

## 1. Introduction

To fulfil the increasing electrical energy demand of daily life, the development of extremely economical and efficient energy storage devices is desperately required. Among the different types of energy storage devices, supercapacitors (SCs) are one of the most effectively applied in the fields of portable electronics, telecommunications, back-up power systems, vehicles and so on, for storing intermittent electrical energy.^1–4^ Supercapacitors are mostly classified into two categories, depending on the charge storage mechanism. The first type is the pseudocapacitor, also known as the faradaic supercapacitor, which makes use of quick and reversible redox reaction to accumulate charges. The other type is the electrical double-layer capacitor (EDLC), whose electrode material is primarily made up of carbon-based conducting porous materials. Various kinds of EDLC materials have been employed as SCs electrodes such as graphene, carbon nanotubes (CNTs), carbide-derived carbons, zeolite-templated carbon, carbon-nanofibers and activated carbon.^5–7^ Among these materials, the most widely utilized in asymmetric devices is activated carbon as a result of its ease of production, high specific area, good porosity, relatively low cost, and light weight.^8^

Generally, the pseudocapacitors operating by the Faraday process possess much more specific capacitance as compared to EDLCs.^9,10^ Over the past few years, transition metal oxides have been significantly engaged as supercapacitor electrode materials. For supercapacitor applications, working principle of transition metal oxides is mainly based on the rapid faradaic redox reactions.^11–16^ One of the extensively investigated transition metal oxides, zinc oxide (ZnO) is well-recognized active material with high energy density. Additionally, ZnO possesses excellent electrical conductivity, which is significantly greater than other metal oxide materials.^17,18^ When molybdenum (Mo) is doped into ZnO, it become quite attractive due to valence difference of 4 between Mo^6+^ and Zn^2+^. The ionic radius of Zn^2+^ is 0.074 nm, and that of Mo^6+^ is 0.062 nm; as a result it can be theoretically possible for Mo^6+^ to substitute Zn^2+^ in the ZnO film. Accordingly, doped zinc oxide might be anticipated to have a promising prospect.^19,20^

The purpose of this investigation is to improve the electrochemical properties of ZnO-based metal oxides by incorporating high-capacity molybdenum. This study presents a facile KOH-produced hydrothermal method followed by short duration thermal calcination for the synthesis of molybdenum-doped zinc oxide (Mo:ZnO) nanoflakes on nickel (Ni)-foam. Over the synergistic contribution from each transition metal, the Mo:ZnO nanoflakes demonstrated wide potential window, high specific capacitance, and long-term cycling performance. Moreover, asymmetric supercapacitor based on Mo:ZnO and activated carbon on Ni-foam (AC@NF) showed a superior energy density, excellent cycling stability, and high specific capacitance.

## 2. Experimental

### 2.1 Materials

All chemicals were consumed as received. Zinc nitrate hexahydrate (Zn(NO_3_)_2_·6H_2_O, 98%) and sodium molybdate dihydrate (Na_2_MoO_4_·2H_2_O, 99.5%) were purchased from Sigma-Aldrich and urea was procured from Xilong Chemical Co., Ltd., China. Hydrochloric acid and NF was purchased from Sinopharm Chemical Reagent Co., Ltd. and from MTI Corp., respectively. A strip of NF with dimension of 1 × 3 cm, was cleaned in 3 M HCl, deionized water and absolute ethanol by sonication before drying overnight at 60 °C.

### 2.2 Preparation of electrode materials

**Fig. 1:**
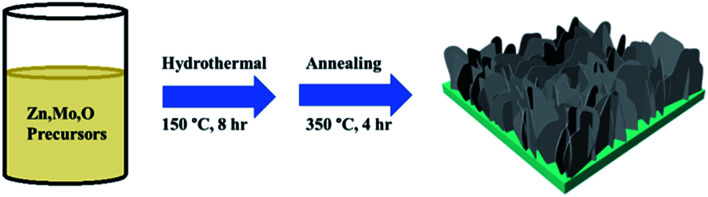
Schematic illustration of the synthesis process of Mo:ZnO nanoflakes.

Mo:ZnO nanoflakes were grown on NF using single-step hydrothermal process. Zn(NO_3_)_2_·6H_2_O (1 mmol), CH_4_N_2_O (2 mmol) and Na_2_MoO_4_·2H_2_O (1-3 wt%) were dissolved in DI water (40 mL). The obtained solution was stirred for 1 h. The strip of NF and Mo:ZnO precursor solution were then transferred to Teflon-lined autoclave (50 mL). The autoclave was heated for 8 h, at 160 °C. After 8 h, the heating was stopped and autoclave was gradually cooled to room temperature. The NF was washed with DI water as well as ethanol to get rid from the unreacted residues. The obtained solids were dried for 12 h in a vacuum oven, at 80 °C. Finally, the samples were calcined in air for 4 h, at 350 °C. Fig. 1 presents a schematic diagram of the synthesis of a Mo:ZnO nanoflakes supercapacitor electrode induced by single-step hydrothermal process. The calculated mass loading of active material on NF was 2 mg cm^−2^. For a comparative study, ZnO was prepared by same procedure.

### 2.3 Characterization

The morphology of as-prepared electrode was analyzed by scanning electron microscopy (S-4800 Hitachi) and high-resolution transmission electron microscopy (FEI Tecnai G2 F20) at an acceleration voltage of 200 kV. X-ray diffraction (XRD, PANalytical, X'Pert-PRO MPD) using Cu Kα radiation was used to check the crystallinity of the as-prepared samples. X-ray photoelectron microscopy (XPS, Thermo Scientific, K-Alpha) using Al Kα monochromatized radiation was used to analyze the elemental composition. Three-electrode setup (Autolab PGSTAT 302N) used to examine the electrochemical performance of the as-prepared electrode. The counter electrode (platinum), reference electrode (Ag/AgCl), and the as-prepared electrode was used as a working electrode in three electrode system. 3 M KOH was used as an electrolyte for examined all electrochemical performances.

### 2.4 Fabrication of Mo:ZnO @NF//AC@NF based asymmetric supercapacitor

The device was assembled using AC@NF and Mo:ZnO@NF as a negative and positive electrode, respectively. AC electrode was prepared by using activated carbon, mesoporous carbon and Nafion in the ratio of 80 : 15 : 5 respectively to coated NF. The laboratory filter paper was used as a separator and the device was prepared using a split test cell.

## 3. Results and discussion

### 3.1 Structure and surface morphology

**Fig. 2:**
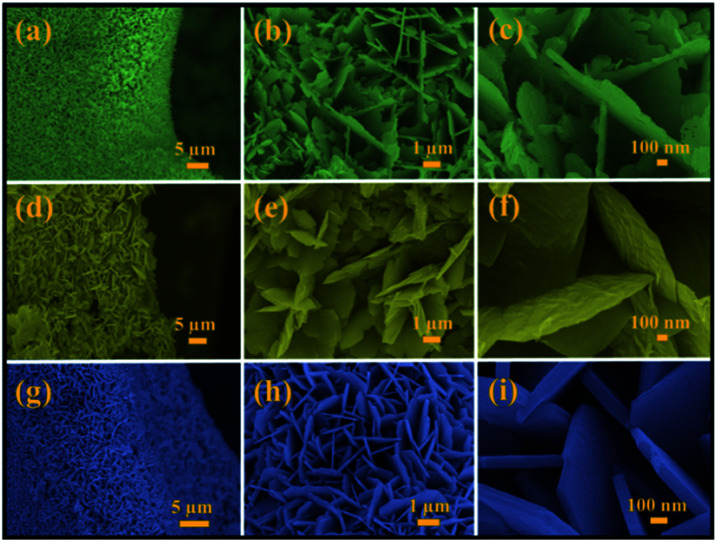
SEM images of Mo:ZnO nanoflakes at different magnifications: (a–c) 0.25 wt% of KOH (d–f) 0.5 wt% of KOH and (g–i) 1 wt% of KOH.

Mo:ZnO was synthesized using a single step hydrothermal reaction. The Mo:ZnO particles were aggregated as well as self-assembled on the NF as substrate. NF substrate was acted as interconnecting network template. The morphology of the Mo:ZnO was evaluated by SEM. Fig. 2 shows the SEM images of Mo:ZnO nanoflakes at different KOH concentrations. The morphology as well as dimension of Mo:ZnO nanoflakes was similar at different KOH concentrations. Individual nanoflake exhibited approximately thickness of 100 nm. It was noted that Mo:ZnO nanoflakes were grown uniformly and almost perpendicular to the NF substrate, in the hydrothermal reaction of 8 h. Clearly, the KOH concentration within the hydrothermal environment found to have intense influence on the surface structures.^21^ As a result of the anisotropic growth process, the Mo:ZnO nanoflakes were attached to each other and created a uniform array over a large area on NF. The network of interconnected Mo:ZnO nanoflakes arrays was retained in different KOH concentration. Such a well interconnected network found supportive for fast ion diffusion and efficient electron transport.^22^ Fig. S1† presents the morphology of the as-synthesized ZnO at low and high magnification under same conditions. ZnO exhibited flower like morphology.

**Fig. 3:**
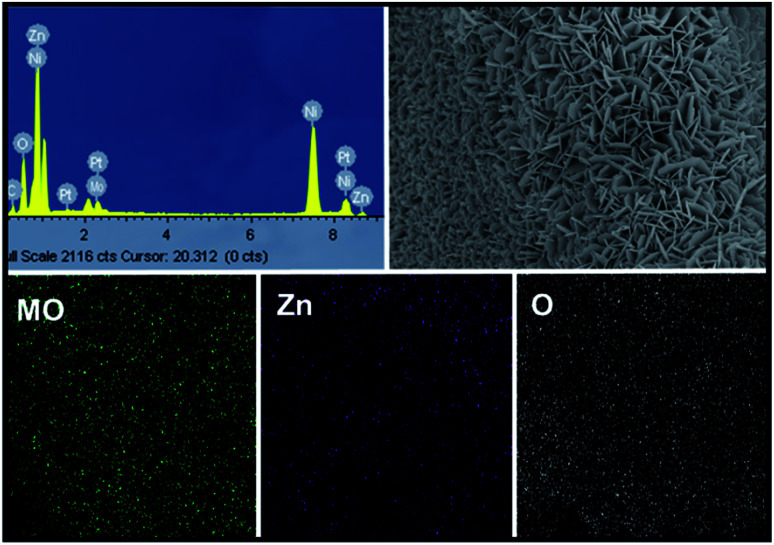
EDX pattern and SEM elemental mapping of the Mo:ZnO nanoflakes.

**Fig. 4:**
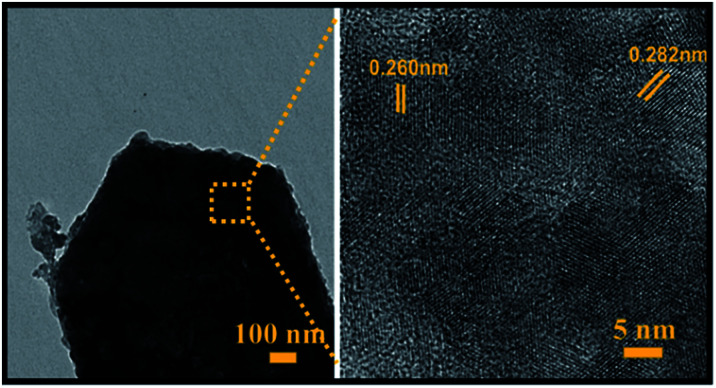
HRTEM images of a Mo:ZnO nanoflakes at different magnifications.

Moreover, SEM elemental mapping and EDX pattern of Mo:ZnO nanoflakes showed the signal of Zn, Mo and O. The results confirmed the uniform distribution of Zn, Mo and O as shown in Fig. 3. To reveal the formation process in more detail, the Mo:ZnO nanoflakes were analysed by high-resolution TEM (HRTEM), obtained results are shown in Fig. 4. The nanoflakes-types Mo:ZnO (detached from NF) confirmed the width of 800–1000 nm and lattice spacing was observed from 0.260 to 0.282 nm that corresponded to the reported in the literature.^23^

**Fig. 5:**
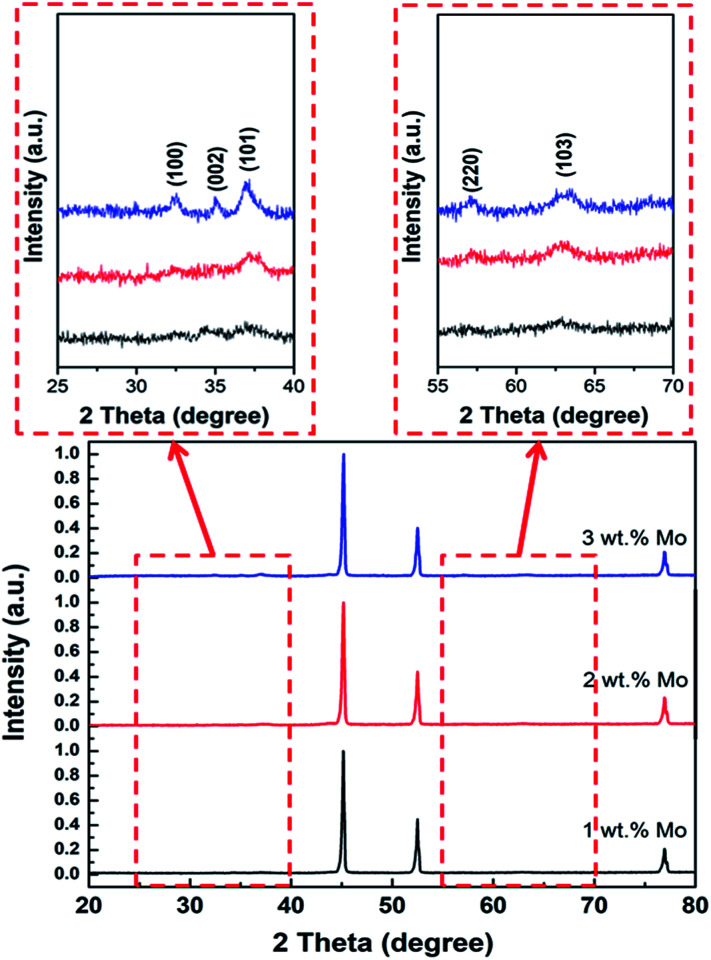
XRD patterns of Mo:ZnO nanoflakes.

Fig. 5 shows the XRD peaks of the Mo:ZnO nanoflakes with different weight concentration of Mo. The diffraction peak at 2*θ* = 34.2° correspond to (002) crystal plane shifted towards higher value with increasing Mo concentration in Mo:ZnO nanoflakes, indicated reduction of inter-planar spacing “*d*” in the films. For 1 wt% Mo doping sample, the intensity of (002) crystal phase at 2*θ* = 34.2° was low as compared with that of the (103) crystal phase at 2*θ* = 62.8°. Also, the crystallographic (002) phase became broad with increased in Mo concentration. The intensity of (002) crystal phase found still lower than (103) crystal phase, when 2 wt% Mo doping was used. However, the XRD peaks position of 3 wt% Mo doping sample exactly matched with JCPDS 01-079-0207. The (002) crystal phase of 3 wt% Mo doped ZnO sample indicated that the Mo:ZnO film was crystallized in hexagonal wurtzite phase.^23^ Due to appropriate doping, we used 3 wt% Mo doped ZnO for further characterization and electrochemical performances.

**Fig. 6:**
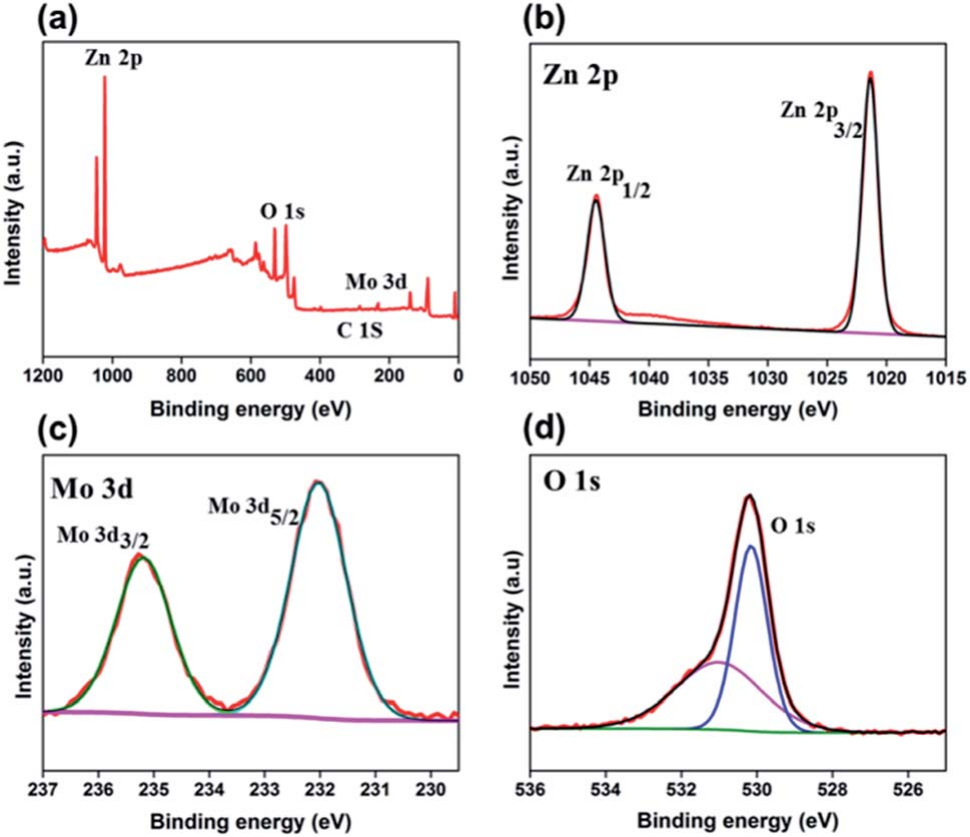
XPS survey spectrum (a) and core level spectra of Zn2p (b), Mo3d (c), and O 1s (d) for Mo:ZnO nanoflakes.

XPS was carried out to investigate the chemical valence states of the different elements and surface elemental composition in 3 wt% Mo doped ZnO. Fig. 6a presents the XPS survey spectrum, showed the presence of Mo, Zn, O as well as C in the as-prepared Mo:ZnO sample. The peaks for C in the spectrum might be due to CO_2_, adsorbed on the sample surface and the adventitious hydrocarbon present in the XPS instrument itself. Fig. 6b presents the Zn2p spectrum. The peak of binding energy at 1022.7 eV was assigned to Zn2p_3/2_ peak of the Zn^2+^ and at 1045.5 eV was assigned to Zn2P_1/2_ peak of the Zn^2+^. Metallic Zn with a binding energy of 1021.50 eV was not observed, which confirmed that Zn exists only in the oxidized state.^24^ The Mo3d XPS spectrum (Fig. 6c) showed two peaks at approximately 232.15 eV and 235.25 eV corresponding to Mo3d_5/2_ and Mo3d_3/2_, respectively.^25^Fig. 6d shows the O 1s XP spectrum, the binding energy at 530.8 eV was assigned to O 1s.^26^

### 3.2 Electrochemical performance

**Fig. 7:**
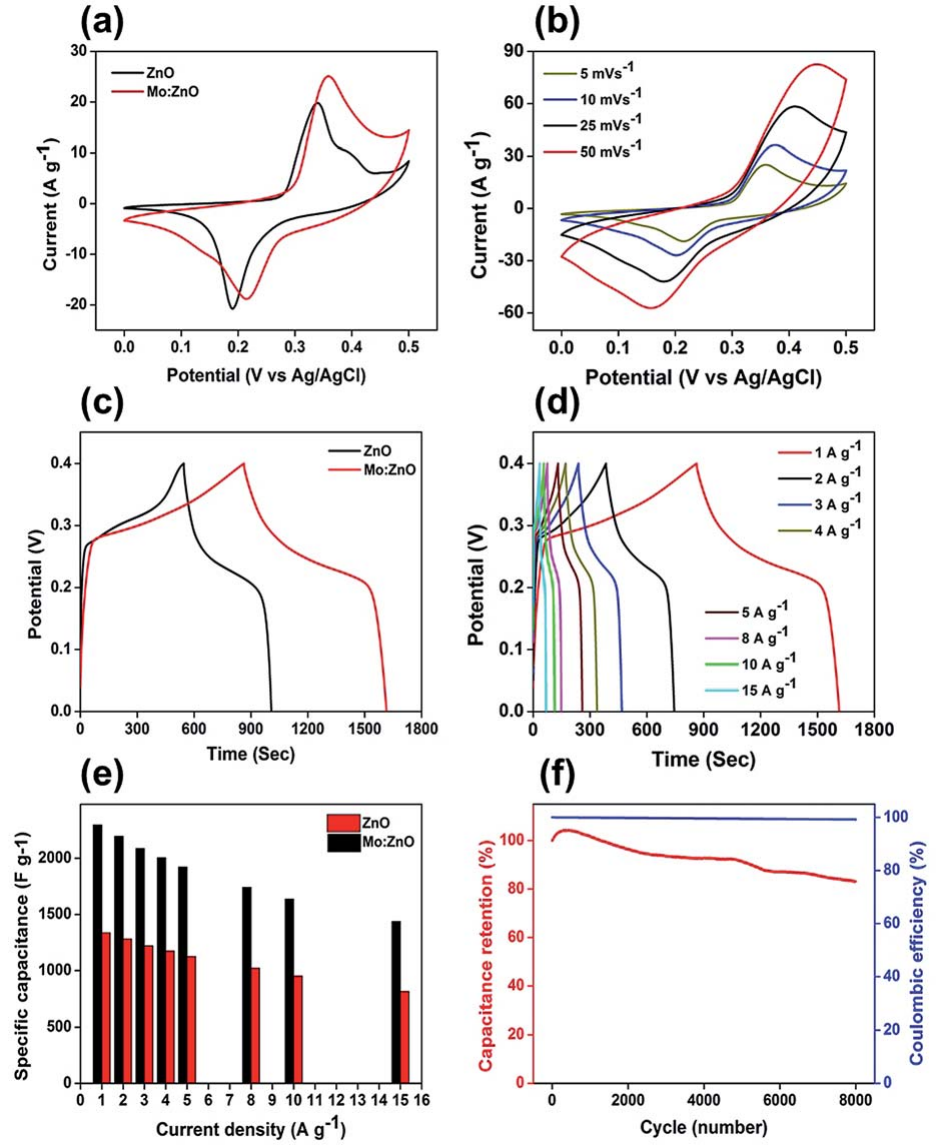
Electrochemical performance of Mo:ZnO and ZnO (a) CV curves at a scan rate of 5 mV s^−1^, (b) CV curves of Mo:ZnO at different scan rates, (c) charge/discharge curves at current density of 1 A g^−1^, (d) charge/discharge of Mo:ZnO at different current densities, (e) rate capability of Mo:ZnO and ZnO, and (f) cycling stability and coulombic efficiency for 8k cycles.

The electrochemical performance of the 3 wt% Mo:ZnO and ZnO electrodes was investigated in a three electrode configuration. The results obtained from the cyclic voltammogram (CV) curves illustrated the oxidation/reduction behaviour of the electrode materials, which confirmed the faradaic redox reaction. Fig. 7a shows typical CV of the Mo:ZnO and ZnO electrode at scan rate of 5 mV s^−1^. Mo:ZnO showed a higher area under the curve than ZnO, proposing that a higher amount of energy was stored on the Mo:ZnO electrode at electroactive sites as compared to ZnO.^27^Fig. 7b reveals the CV curves of Mo:ZnO electrode at a scan rate from 5 to 50 mV s^−1^. At higher scan rates, the surface reaction was observed lower because of the limited time. On the other hand, the ions had sufficient time to react and caused to enhance the charge storage at low scan rates. These results showed that the specific capacitance at low scan rates was high.^28^Fig. 7c shows the galvanostatic charge–discharge (GCD) profiles of the Mo:ZnO and ZnO electrode observed at a current density of 1 A g^−1^. The charge–discharge performance of Mo:ZnO was found to be better than ZnO. Mo:ZnO electrode revealed a much longer charge–discharge time than ZnO electrode and possessed high specific capacitance. This phenomenon could be due easy electron mobility and high electrical conductivity provided by Mo to ZnO in Mo:Zn electrode resulted in excellent specific capacitance.^29,30^Fig. 7d presents the detailed GCD profiles of Mo:ZnO electrode, which were obtained at different current densities from 1 A g^−1^ to 15 A g^−1^ in the potential range from 0 to 0.4 V. The GCD curves revealed the non-linear shapes which attributed to the characteristics of the faradaic redox reaction of pseudocapacitance behaviour of the Mo:ZnO electrode and in consistent with the redox peaks of CV curves.^31^ Furthermore, the GCD curves were observed to be same, indicated that the redox reaction was reversible in nature and the Mo:ZnO electrode had better capacitive properties.^28^ Non-linear behaviour was observed in the discharge curves. Therefore, *C*_s_ was calculated by means of the following equation:^32^1
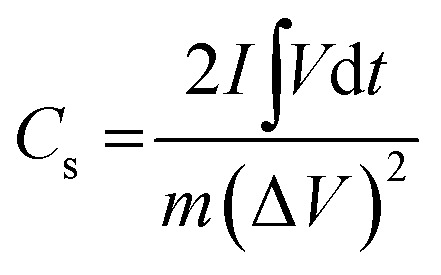
where *I* represents the current (A) of active material, *V* represents the potential (V) of active material, *m* represents the mass (g) of active material and 
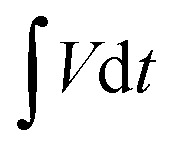
 represents the area under the discharge curve.

A maximum specific capacitance of 2296 F g^−1^ was observed, when a current density of 1 A g^−1^ was used. The specific capacitance was calculated to be 2196, 2088, 2005, 1923, 1741, 1636 and 1438 F g^−1^ at current densities of 2, 3, 4, 5, 8, 10, and 15 A g^−1^, respectively (Fig. 7e). The performance of the specific capacitance can be associated to the faster transport of ions in the electrode/electrolyte interface. This was predominantly as a result of high surface area, which caused fast migration of the ions in and out of the interface. The accessibility of ions at higher current density was lower because of the limited period for full contact made a sudden specific capacitance drop, when the current density was increased from 1 to 15 A g^−1^. The electrochemical stability of the Mo:ZnO electrode was tested for 8000 GCD cycles at a constant current density of 10 A g^−1^ (Fig. 7f). At first, the value of *C*_s_ increased from 0 to 500 GCD cycles as a result of activation process.^31^ After 8000 GCD cycles, the capacitance retention was observed 88% of the initial value which confirmed the good cycling stability of Mo:ZnO. Moreover, the coulombic efficiency, which defined the efficient transport of electrons facilitating in the faradaic redox reaction in the electrode/electrolyte surface, yielded to 99.9% over 8000 cycles.

Table S1† lists the supercapacitor performance of Mo:ZnO and ZnO-based supercapacitor electrode materials. The improvement in the performance of Mo:ZnO electrode was owing to the following reasons: first, the electrode materials deposited directly on NF avoid the dead volume caused by binder. Second, the electrode material directly deposited on NF had high interfacial contact between the electrode material and substrate, which enhanced electron transport. Third, the uniformly grown nanoflakes on NF offered an effective path for ion and electron transport, which improved the specific capacitance and rate capability. Fig. S2† displays Nyquist plots for Mo:ZnO and ZnO electrode materials which carried out in KOH electrolyte (3 M) over the frequency in the range of 0.01 Hz to 10 MHz. An intercept at the high frequency region with the real part (Zr) was attributed to the equivalent series resistance (ESR). Mo:ZnO and ZnO electrode materials showed an ESR of 0.70 and 1.07 Ω, respectively that pointed to a small ohmic loss during discharge. The absence of semicircles showed that there was no charge-transfer resistance in the high frequency region. The Warburg impedances (*W*) for the Mo:ZnO, and ZnO electrodes were 25.5, and 25.6 Ω, respectively. *W* is associated with the diffusion of OH^−^ ions produced by the reaction in the electrolyte.^33^

### 3.3 Performance of the asymmetric Mo:ZnO//AC device

**Fig. 8:**
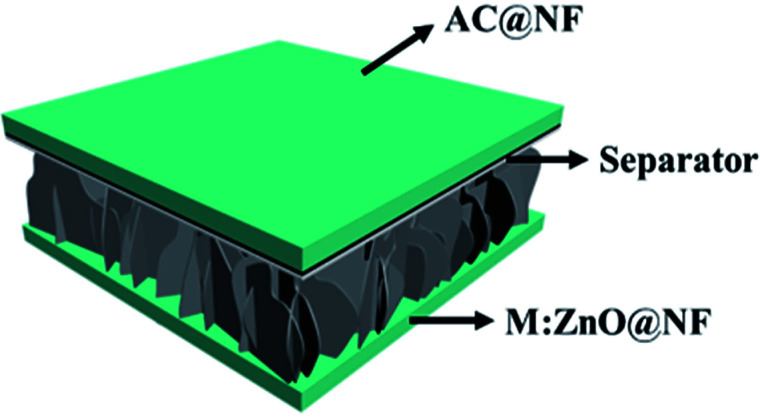
Schematic diagram of the ASC Mo:ZnO//AC supercapacitor device.

Owing to the superior performance of Mo:ZnO, the energy storage performance of the Mo:ZnO nanoflakes electrode were assessed for practical applications. The asymmetric supercapacitors were assembled using Mo:ZnO as the positive electrode and AC as the negative electrode (Fig. 8). The mass of AC for the negative electrode was calculated using the following equation to obtain charge balance on both electrodes.^34^2
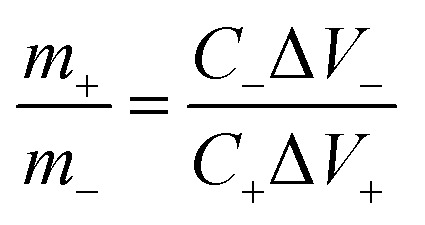
where *m*_+_ represents mass loading of positive (Mo:ZnO@NF) electrode, *C*_+_ represents *C*_s_ of positive (Mo:ZnO@NF) electrode and Δ*V*_+_ represents potential window of the positive (Mo:ZnO@NF) electrode. Similarly, *m*_−_ represents mass loading of negative (AC@NF) electrode, *C*_−_ represents *C*_s_ of negative (AC@NF) electrode and Δ*V*_−_ represents the potential window of the negative (AC@NF) electrode.

**Fig. 9:**
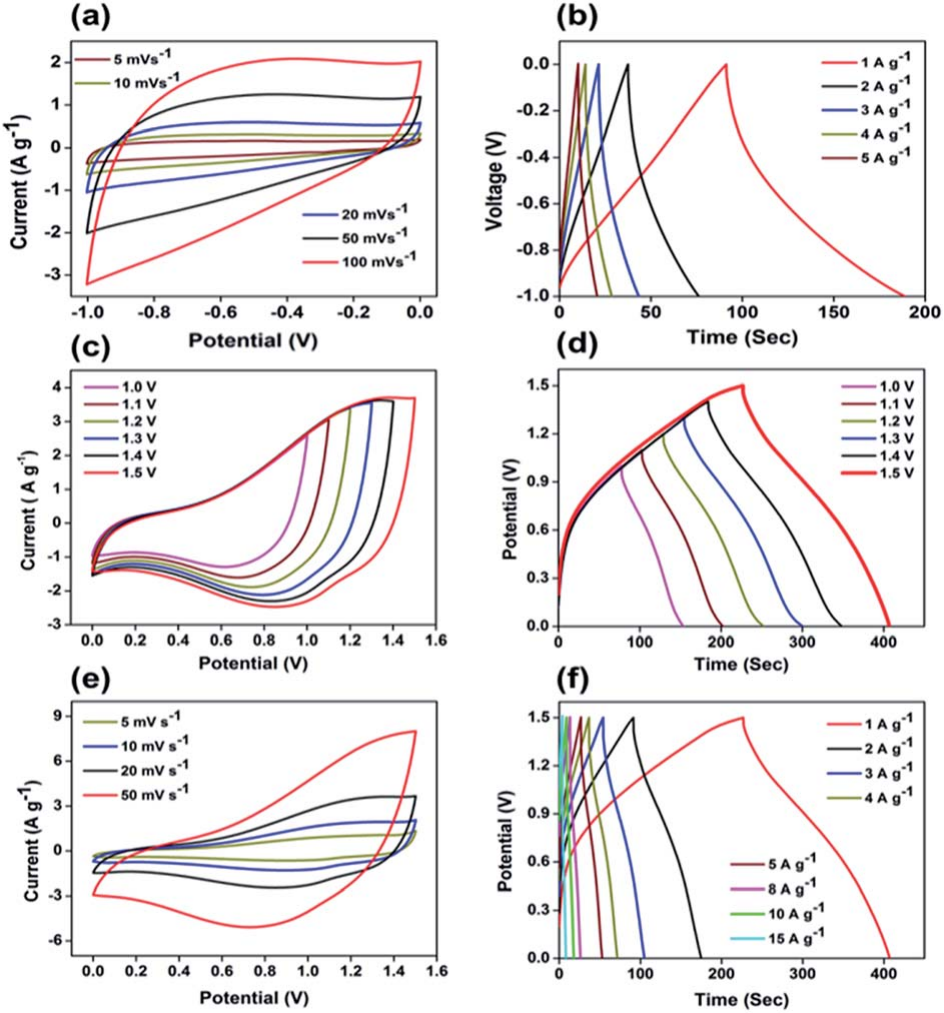
Electrochemical performance of the ASC: (a) CV curves of AC@NF at different scan rates, (b) galvanostatic charge/discharge profiles at different current densities ranging from 1 to 5 A g^−1^ within the potential window of −1.0 to 1 V, (c) CV curves for different potential windows measured at a scan rate of 20 mV s^−1^, (d) galvanostatic charge–discharge curves for different potential windows measured at a current density of 1 A g^−1^, (e) CV curves at different scan rates for 0 to 1.5 V, and (f) galvanostatic charge/discharge curves for a fixed potential window from 0 to 1.5 V measured at different current densities.

In order to evaluate the supercapactive performance of Mo:ZnO@NF//AC@NF (ASC), 3 M KOH was used. The mass loading of Mo:ZnO@NF//AC@NF (ASC) was 21 mg cm^−2^. By comparing the positive electrode and negative electrode at 10 mV s^−1^, it can be concluded that Mo:ZnO@NF electrode material can work at a positive potential (*vs.* Ag/AgCl) over the electrochemical reaction (Fig. S3†). In contrast, an almost rectangular CV curve was observed for AC@NF within the potential window of 0 V to −1.0 V, attributed the charge storage occurred by means of the simple electrosorption of electrolyte ions on electrode surface. Fig. 9a presents CV curves of AC@NF at different scan rates ranging from 5 to 100 mV s^−1^. Fig. 9b shows the GCD profiles of the AC@NF at different current densities. The negative electrode showed a capacitance of 133 F g^−1^ at current density of 1 A g^−1^. In view of the working potential of Mo:ZnO@NF and AC@NF, cell with voltage of 1.5 V was employed to test the ASC. The CV curve for the ASC at a fixed scan rate of 20 mV s^−1^ and at different voltages is presented in the Fig. 9c. It can be seen that the area under the CV curve increases with rising the potential window, based on CV curves. The best operating potential window was chosen to be 1.5 V which was supported by GCD curves. Similarly, the ASC was also exposed to GCD at different voltage windows at a fixed current density of 1 A g^−1^ (Fig. 9d). The symmetrical nature of the charge–discharge curves indicated the highly capacitive behavior of the ASC. In the case of GCD curves, the voltage followed similar path from 1.0 V to 1.5 V and become steady at 1.5 V. Furthermore, the Mo:ZnO@NF//AC@NF ASC's electrochemical performance was analyzed using different scan rates, ranging from 5 mV s^−1^ to 50 mV s^−1^ as shown in Fig. 9e. As a result of the double layer capacitance of AC@NF and the pseudocapacitance behavior of the Mo:ZnO@NF electrode, the ASC demonstrates the combined contribution from non-faradaic and faradaic reactions. Corresponding variation in the GCD curves can be seen in the Fig. 9f. The measured specific capacitance (*C*_s_) for ASC attained a maximum values of 125.2 F g^−1^ at lowest current density of 1 A g^−1^. Eqn (1) was used to calculate the capacitance of the ASC, where *m* represents the total mass of both electrodes.

**Fig. 10:**
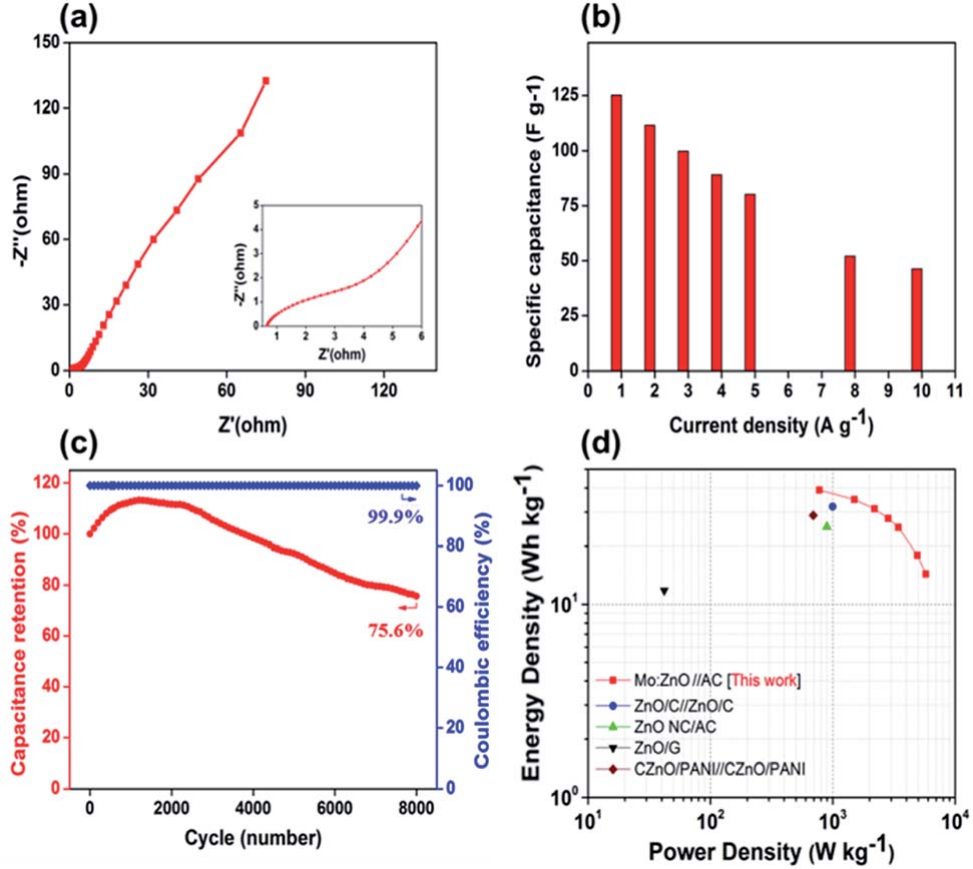
(a) Nyquist plot, (b) rate capability of the ASC, (c) cycling stability and coulombic efficiency, and (d) Ragone plot to compare with the literature, and the Mo:ZnO//AC ASC.

As can be observed in Fig. 10a, the EIS spectra of a Mo:ZnO@NF//AC@NF ASC revealed an internal resistance of 0.65 Ω, which was appropriate for ASC. As depicted in Fig. 10b, the specific capacitance for an ASC reaches a maximum value of 125.2 F g^−1^ at 1 A g^−1^ and decreases to 46.2 F g^−1^ at a current density of 10 A g^−1^. Decrease in specific capacitance was owing to the ineffectiveness of the electroactive sites of the electrodes to maintain the redox transitions. As shown in Fig. 10c, the Mo:ZnO@NF//AC@NF ASC exhibited coulombic efficiency of 99% and exceptional capacitive retention of 75.6% after 8000 GCD cycles. The power density (*P*_d_, W kg^−1^) and energy density (*E*_d_, W h/kg) were estimated using following equations:^35,36^3
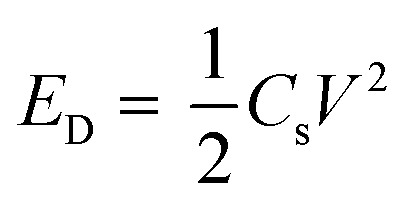
4
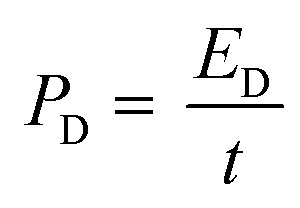
where *I* is current, *V* is potential, and *m* is mass of active material. A Ragone plot was applied to describe the ability of an energy storage devices, in which the energy density was calculated as a function of the power density. The Ragone plot of the different ZnO-based SCs is depicted in Fig. 10d. The Mo:ZnO@NF//AC@NF ASC presented a maximum power density of 7.425 kW kg^−1^ (at 8.2 W h/kg) and a maximum energy density of 39 W h/kg (at 778 W kg^−1^). These results obtained were remarkably greater than any other reported ZnO-based SCs. Exceptional performance is observed for the Mo:ZnO@NF//AC@NF ASC of all the ZnO-based supercapacitors examined in the literature (Table S2†).

## 4. Conclusion

In summary, an effective approach for the fabrication of Mo:ZnO supercapacitor electrodes was presented. The resulting Mo:ZnO electrode with a nanoflakes type morphology exhibited good electrochemical capacitance performance with a high energy and power density. Moreover, the capacitance retention was 75.6% after 8000 charge/discharge cycles, indicating the excellent cycling stability of the ASC. The performance of the electrodes could be enhanced further by optimization with different substrates and electrolytes. These results suggested that Mo:ZnO was a promising electrode material for high-efficiency supercapacitors and that the scope of Mo:ZnO-based electrodes might be expanded greatly to other efficient energy storage systems.

## Conflicts of interest

There are no conflicts to declare.

## Supplementary Material

RA-009-C9RA05051E-s001
