# Supplementary material for: Mo-doped ZnO nanoflakes on Ni-foam for asymmetric supercapacitor applications
Source: RSC Adv. 2019 Sep 2;9(47):27432–8. doi: 10.1039/c9ra05051e (PMC9070622; doi:10.1039/c9ra05051e)
Supplement: RA-009-C9RA05051E-s001 [file RA-009-C9RA05051E-s001.pdf]

Electronic Supplementary Material (ESI) for RSC Advances.
This journal is © The Royal Society of Chemistry 2019

Supplementary Material

Mo-doped ZnO nanoflakes on Ni-foam for asymmetric supercapacitor applications

Awais Ali [a], Muhammad Ammar [a,b,*], Muddassir Ali [c], Zaid Yahya [a], Muhammad Yasar Javaid [d], Sadaf ul Hassan [e], Toheed Ahmed [b,f]

[a] Department of Chemical Engineering Technology, Government College University, Faisalabad 38000, Pakistan.

[b] University of Chinese Academy of Sciences, Beijing, 100049, China.

[c] Department of Energy Engineering, Faculty of Mechanical and Aeronautical Engineering, University of Engineering and Technology, Taxila 47080, Pakistan.

[d] Department of Mechanical Engineering Technology, Government College University, Faisalabad 38000, Pakistan.

[e] Department of Chemistry, COMSATS University, Islamabad, Lahore Campus 45550, Pakistan.

[f] Department of Applied Chemistry, Government College University, Faisalabad 38000, Pakistan.

* Corresponding author: mammar@gcuf.edu.pk (M. Ammar), Tel/Fax: +92419203027

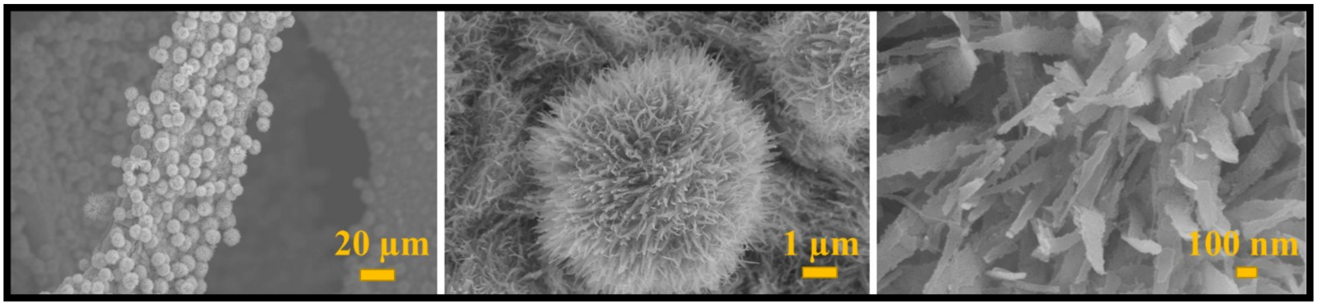

Fig. S1 SEM images of ZnO at different magnifications.

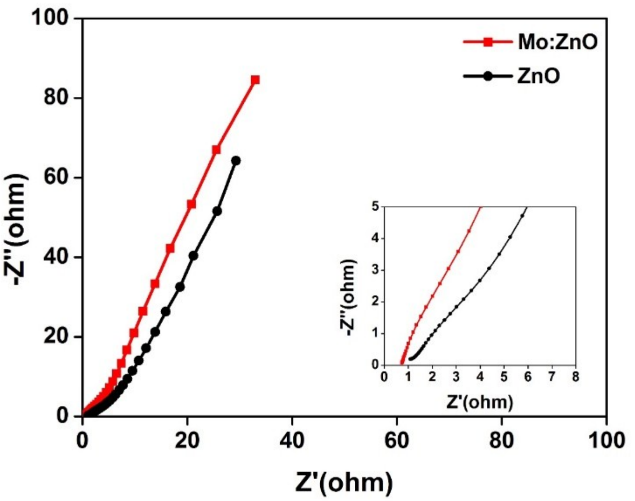

Fig. S2 Nyquist plot of Mo:ZnO and ZnO in 3M KOH.

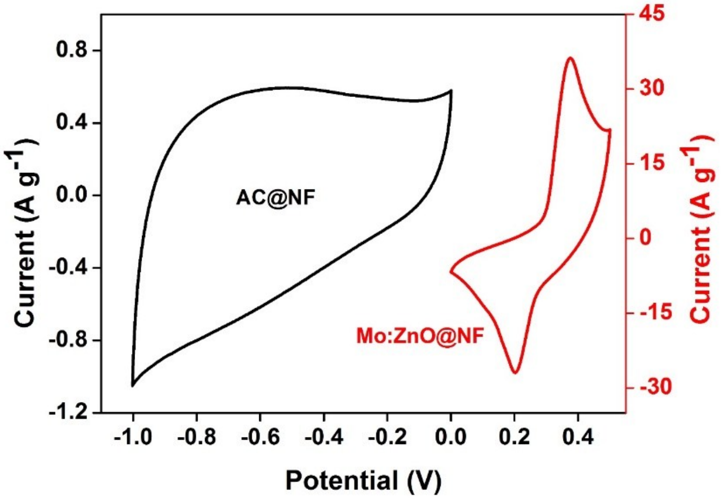

Fig. S3 CV curves of AC@NF and Mo:ZnO in a 3M KOH electrolyte at 10 mV s^{-1} scan rate.

Table S1. Comparison of several metal oxide reported as supercapacitor electrode materials in the literature and this study

Electrode	Electrolyte	Current density	Scan rate (mV s^{-1})	Specific capacitance/ Capacity	Ref.
G-ZnO@GC	6 M KOH	-	5	122.4 F g^{-1}	1
ZnO@GF	2 M KOH	1 A g^{-1}	-	400 F g^{-1}	2
G-ZnO	1 M KCl	-	2	146 F g^{-1}	3
ZnO/rGO	1M Na_2SO_4	-	10	95 F g^{-1}	4
3D G-ZnO	1 M KOH	-	5	554 F g^{-1}	5
ZnO-MnO_2	1M Na_2SO_4	0.5 A g^{-1}	-	423 F g^{-1}	6
ZnOAu-MnO_2	0.1M Na_2SO_4	2.6 A g^{-1}	-	478 F g^{-1}	7
MnO_2-ZnO	0.1M Na_2SO_4	-	2	571 μF cm^{-2}	8
Core-shell ZnO-NiO	1 M KOH	5 mA cm^{-2}	-	4.1 F cm^{-2}	9
ZnO-NiO	1 M KOH	5 mA cm^{-2}	-	0.5 F cm^{-2}	9
ZnO-NiO	3 M KOH	-	5.8	649 F g^{-1}	10
Mo:ZnO	3 M KOH	1	-	2,296 F g^{-1}	This work

Table S2. Comparison with other Zinc oxide-based supercapacitor devices

Electrode	Electrolyte	Current density/scan rate	Specific capacitance	Capacitance retention	Energy density @ Power density	Ref.
ZnO/C//ZnO/C	1M Na_2SO_4	2.5 A g^{-1}	92 F g^{-1}	92% after 400 cycles	32 Wh kg^{-1}@1000 W kg^{-1}	[11]
ZnO NC//AC	1M KOH	1.33 A g^{-1}	126 F g^{-1}	62.5% after 5,000 cycles	25.2 Wh kg^{-1}@896.4 W kg^{-1}	[12]
MRGO/ZnO	1M Na_2SO_4	1 A g-1	201 F g^{-1}	93% after 3,000 cycles	4.33 W h kg^{-1}@8.3 W kg^{-1}	[13]
G/ZnO	1M KOH	10 mV s^{-1}	236 F g^{-1}	90% after 200 cycles	11.8 W h kg^{-1}@42 W kg^{-1}	[14]
CZO/PANI//CZO/PANI	1M H_2SO_4	-	-	-	28.88 W h kg^{-1}@694 W kg^{-1}	[15]
Mo:ZnO@NF//AC@NF	3M KOH	1 A g^{-1}	125 F g^{-1}	75% after 8,000 cycles	39 Wh kg^{-1}@778 W kg^{-1}	This work

References

1. M. Saranya, R. Ramachandran and F. Wang, *Journal of Science: Advanced Materials and Devices*, 2016, **1**, 454-460.
2. X. Dong, Y. Cao, J. Wang, M. B. Chan-Park, L. Wang, W. Huang and P. Chen, *RSC Advances*, 2012, **2**, 4364-4369.
3. T. Lu, L. Pan, H. Li, G. Zhu, T. Lv, X. Liu, Z. Sun, T. Chen and D. H. Chua, *Journal of Alloys and Compounds*, 2011, **509**, 5488-5492.
4. I. Y. Bu and R. Huang, *Materials Science in Semiconductor Processing*, 2015, **31**, 131-138.
5. X. Li, Z. Wang, Y. Qiu, Q. Pan and P. Hu, *Journal of Alloys and Compounds*, 2015, **620**, 31-37.
6. M. Huang, F. Li, X. L. Zhao, D. Luo, X. Q. You, Y. X. Zhang and G. Li, *Electrochimica Acta*, 2015, **152**, 172-177.
7. Y. Zhao and P. Jiang, *Colloids and Surfaces A: Physicochemical and Engineering Aspects*, 2014, **444**, 232-239.
8. X. Zhou and L. Ma, *Thin Solid Films*, 2015, **597**, 44-49.
9. C. Wei, H. Pang, C. Cheng, J. Zhao, P. Li and Y. Zhang, *CrystEngComm*, 2014, **16**, 4169-4175.
10. H. Pang, Y. Ma, G. Li, J. Chen, J. Zhang, H. Zheng and W. Du, *Dalton Transactions*, 2012, **41**, 13284-13291.
11. C. Sasirekha, S. Arumugam and G. Muralidharan, *Applied Surface Science*, 2018, **449**, 521-527.
12. X. He, J. E. Yoo, M. H. Lee and J. Bae, *Nanotechnology*, 2017, **28**, 245402.
13. Y. Guo, B. Chang, T. Wen, C. Zhao, H. Yin, Y. Zhou, Y. Wang, B. Yang and S. Zhang, *RSC Advances*, 2016, **6**, 19394-19403.
14. E. R. Ezeigwe, M. T. Tan, P. S. Khiew and C. W. Siong, *Ceramics International*, 2015, **41**, 715-724.
15. R. Karthik and S. Thambidurai, *Journal of Materials Science: Materials in Electronics*, 2017, **28**, 9836-9851.
